# A methylomics-associated nomogram predicts the overall survival risk of stage III to IV ovarian cancer

**DOI:** 10.1097/MD.0000000000032766

**Published:** 2023-02-03

**Authors:** Xuan Wei, Wencheng Hu, Kexi Mao

**Affiliations:** a Department of Gynaecology, Taikang Tongji (Wuhan) Hospital, Wuhan, China; b Department of Emergency, Taikang Tongji (Wuhan) Hospital, Wuhan, China.

**Keywords:** DNA methylation, nomogram, ovarian cancer, overall survival, signature

## Abstract

Accumulating studies demonstrated that DNA methylation may be potential prognostic hallmarks of various cancers. However, few studies have focused on the power of DNA methylation for prognostic prediction in patients with stage III to IV ovarian cancer (OC). Therefore, constructing a methylomics-related indicator to predict overall survival (OS) of stage III to IV OC was urgently required. A total of 520 OC patients with 485,577 DNA methylation sites from TCGA database were selected to develop a robust DNA methylation signature. The 520 patients were clustered into a training group (70%, n = 364 samples) and an internal validation group (30%, n = 156). The training group was used for digging a prognostic predictor based on univariate Cox proportional hazard analysis, least absolute shrinkage and selection operator (LASSO) as well as multivariate Cox regression analysis. The internal and external validation group (ICGC OV-AU project) were used for validating the predictive robustness of the predictor based on receiver operating characteristic (ROC) analysis and Kaplan–Meier survival analysis. We identified a 21-DNA methylation signature-based classifier for stage III-IV OC patients’ OS. According to ROC analysis in the internal validation, external validation and entire TCGA set, we proved the high power of the 21-DNA methylation signature for predicting OS (area under the curve [AUC] at 1, 3, 5 years in internal validation set (0.782, 0.739, 0.777, respectively), external validation set (0.828, 0.760, 0.741, respectively), entire TCGA set (0.741, 0.748, 0.781, respectively). Besides, a nomogram was developed via methylation risk score as well as a few clinical variables, and the result showed a high ability of the predictive nomogram. In summary, we used integrated bioinformatics approaches to successfully identified a DNA methylation-associated nomogram, which can predict effectively the OS of patients with stage III to IV OC.

## 1. Introduction

Ovarian cancer (OC) was most common malignancy of all gynecologic cancers in the United States, which has the fifth highest tumor-related mortality in women.^[1]^ In spite of improvement in the therapy of advanced OC, the overall 5-year survival rate remains less than 50%.^[2]^ Most patients are diagnosed at an advanced stage which precludes curative therapy.^[3]^ The staging system determined by the International Federation of Gynecology and Obstetrics is commonly employed to identify prognosis and direct an optimized therapeutic schedule.^[4]^ Whereas, International Federation of Gynecology and Obstetrics stage are considered as significant clinical prognostic factors of OC but are insufficient for the prediction of survival time. Thus, it is a crucial to identify diagnostic classifiers which can reliably stratify OC patients for individualized treatment.

Specific molecular biomarkers have been proved to be involved in prognosis of OC. For example, Zheng *et al* identified a molecular marker associated with OC prognosis using bioinformatics analysis and experiments.^[5]^ Zannoni et al showed that M-CAM expression served as a marker of poor prognosis in epithelial OC.^[6]^ Baekelandt et al revealed that p-glycoprotein expression was a marker for chemotherapy resistance and prognosis in advanced OC.^[7]^ In the investigation for reliable potential hallmarks for prognosis of various cancer, DNA methylation has been proved to be potential prognostic predictor. For example, Fiano et al revealed that DNA methylation in repeat negative prostate biopsies served as a marker of missed prostate cancer.^[8]^ Jouinot et al indicated that DNA Methylation was an independent prognosis biomarker of survival in adrenocortical carcinoma.^[9]^ Schmitz et al showed the capacity of a DNA methylation marker panel via liquid-based cervical scrapes to test cervical carcinoma and its precancerous stages.^[10]^ Zhang et al suggested that DNA methylation mediated silencing of microRNA-874 was a promising diagnosis and prognostic hallmark in breast cancer.^[11]^ DNA Methylation was revealed to be a reversible biological signal which may be potential therapeutic targets.^[12]^ Therefore, the comprehensive analysis of DNA methylation is promising in developing reliable prognostic predictors for individualized therapy and improving patients’ survival time. However, few studies have focused on the power of DNA methylation for prognostic prediction in patients with stage III to IV OC. Building a methylomics-related indicator to predict overall survival (OS) of stage III to IV OC seems very promising.

In this study, we identify a DNA Methylation signature via microarray profiling related to OS in stage III to IV OC. The power of the DNA Methylation signature was tested by Kaplan–Meier analysis, Relative operating characteristic curve (ROC) analysis and the result suggested that the hallmark has potential to improve the care of women with stage III to IV OC. In addition, the result also showed that our nomogram had a high prognostic predicted ability.

## 2. Materials and methods

### 2.1. DNA methylation information of stage III to IV OC patients

The stage III to IV OC DNA methylation information from The Cancer Genome Atlas (TCGA) database that was projected to illumina Human Methylation 450 BeadChip (illumina Inc, CA) and eligible clinical information was achieved with R TCGAbiolinks package.^[13]^ OV-AU project was manually downloaded from International Cancer Genome Consortium (ICGC) database.^[14]^ The DNA methylation levels were defined as β values, computed as M/(M + U + 100), with U representing an unmethylated signal and M representing a methylated signal. Patients were excluded from further analysis if lacking of survival data. The present study comprised of a total of 520 stage III-IV OC patients with 485,577 DNA methylation sites. The 520 patients were clustered into a training group (70%, n = 364 samples) for digging a prognostic predictor and an internal validation group (30%, n = 156) for validating the predictive robustness of the predictor. What’s more, the 93 stage III to IV OC patients of OV-AU project from ICGC database were used as an external validation dataset. Least absolute shrinkage and selection operator (LASSO) analysis was adopted to screen the key methylation sites for exploring predictor of stage III to IV OC patients’ OS. In addition, LASSO analysis was carried out using 1000 iterations via a publicly available R package “glmnet.”^[14]^ Our study didn’t involve human beings or animals, so the approval of the Ethics Committee is not necessary for our study. The Institutional Review Board of Taikang Tongji (Wuhan) Hospital approved the study.

### 2.2. Data processing, normalization and determination of differentially expressed methylation sites

Pre-processing of the raw data was completed for digging a prognostic biomarker of stage III to IV OC. A DNA methylation site would be excluded if its value was no available in any sample. Next, the data was normalized via “betaqn” function from wateRmelon package.^[15]^ Then, the total patients were clustered into recurrence cohort and no recurrence cohort via recurrence status. The standardized beta was transformed to M value in accordance to the formulation: M = log (β/(1-β)). M value was used for eliminating the variance arising from various probes. After that, M value was executed for digging the differentially expressed methylation sites between recurrence and no recurrence group using “dmpFinder” function of minfi package.^[16]^

### 2.3. Generation of methylomics-related signature

We executed univariate Cox proportional hazard analysis to dig the methylation sites importantly (*P* < .05) linked to stage III to IV OC patients’ OS as potential factors. Next, the LASSO Cox regression analysis was implemented across the potential factors for further digging the candidate sites involved in stage III to IV OC patients’ OS. After that, the total candidate sites were mapped to the multivariate Cox regression analysis for unearthing the methylome-related classifier for stage III to IV OC patients’ OS. Finally, the combination of a total 21 DNA methylation sites was used to construct the DNA methylation-based prognostic classifier. Then a risk-score formula was generated based on the 21-DNA methylation signature to compute OS risk score of every sample. Patients were assigned to the high-risk cluster if their prognostic risk scores were larger than the cutoff of the median risk score, whereas low-risk cohort consisted of samples with the risk scores that were less than cutoff value. We performed ROC analysis to evaluate the value of the 21-DNA methylation-based signature. Area under the curve (AUC) value achieved from ROC analysis was employed to weigh the predicted power of the methylome-related classifier for stage III-IV OC patients’ OS by the “survivalROC” package.^[17]^ Kaplan–Meier survival was applied to compare differences in OS between high- and low-risk clusters and Kaplan–Meier curves were achieved through the “survival” package.^[18]^

### 2.4. Gene set variation analysis

To dig the 21-DNA methylation signature-based signaling pathways. We executed single sample gene sets enrichment analysis in accordance to TCGA OC mRNA dataset via gene set variation analysis package.^[19]^ The most crucial pathways positively involved in DNA methylation risk score were assessed. Patients were assigned to the high-risk group if their prognostic risk scores were more than the cutoff of the median risk score, while low-risk cohort comprised of samples with the risk scores that were less than cutoff value. Significance was set as *P* < .05.

### 2.5. Construction of the nomogram

To improve the prognostic discrimination of the 21-DNA methylation signature for stage III to IV OC, a nomogram was established across the “rms” R package. The univariate and multivariate Cox proportional hazard analysis were carried out through the methylation risk score and other clinicopathological factors. Cox proportional hazard models was implemented to compute hazard ratios (HR) as well as corresponding 95% confidence interval (CI). Factors that were significant (*P* ≤ .05) from the multivariate Cox proportional hazard analysis were adopted for the construction of the nomogram across the “rms” R package. C-index, ROC and calibration plot and decision curve analysis were used as indicators to detect the prognostic performance of our nomogram. The outcome of the nomogram was exhibited in the calibrate curve, and the 45° line implied the best prediction.

## 3. Results

### 3.1. Clinical features of the study populations

**Figure 1.**
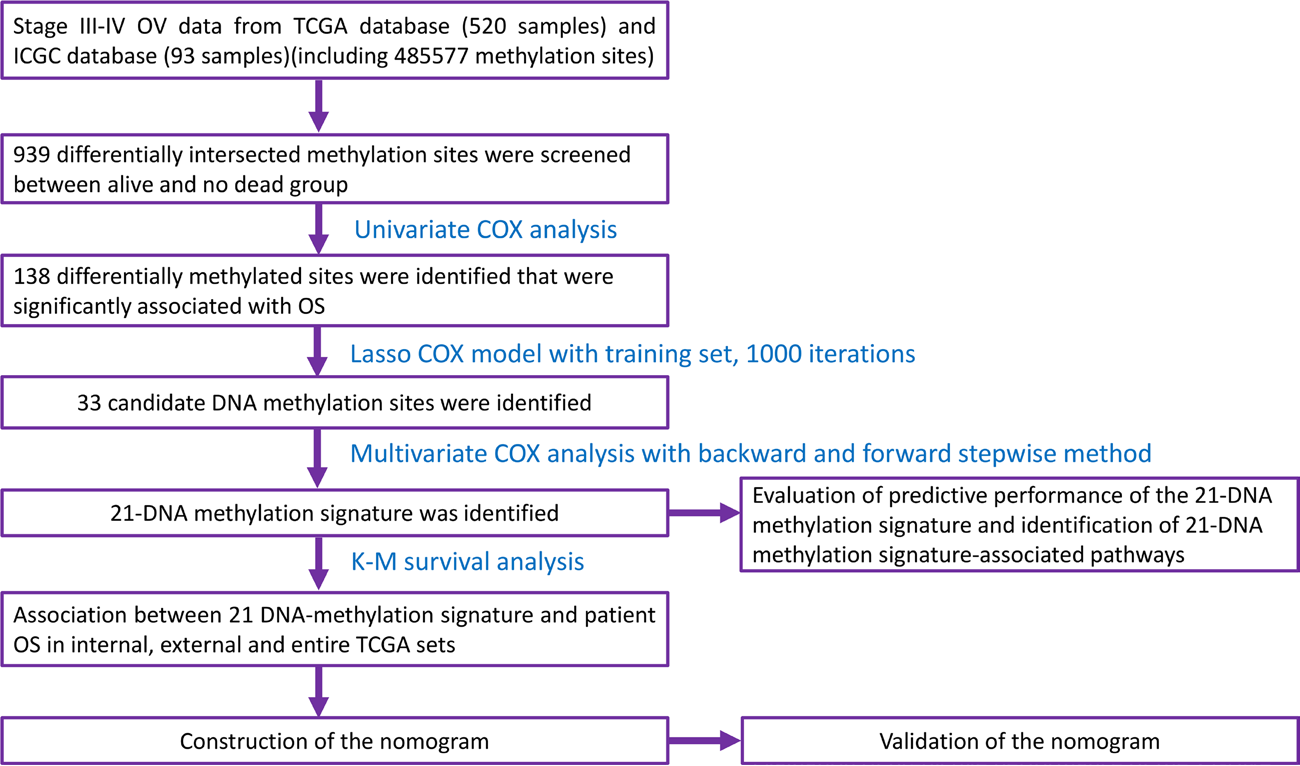
Flow chart of the present study.

Totally, 520 TCGA patients and 93 ICGC database patients who were clinically and pathologically diagnosed as stage III to IV OC were enrolled in present study. The clinical feature of stage III to IV OC patients in TCGA dataset and ICGC dataset was exhibited in Table 1. The experimental procedures were shown in Figure 1.

**Table 1:** Clinical characteristics of patients showed in the study.

Characteristics	Total (n = 520)	Training dataset (n = 364)	Testing dataset (n = 156)	External validation set (n = 93)
**Age**				
<=60	290(55.77)	206(56.59)	84(53.85)	54(58.06)
>60	230(44.23)	158(43.41)	72(46.15)	39(41.94)
**Stage**				
Stage IIIA	8(1.54)	5(1.37)	3(1.92)	79(84.95)
Stage IIIB	23(4.42)	18(4.95)	5(3.21)	
Stage IIIC	403(77.5)	287(78.85)	116(74.36)	
Stage IV	86(16.54)	54(14.84)	32(20.51)	14(15.05)
**Grade**				
G1	6(1.15)	5(1.37)	1(0.64)	
G2	57(10.96)	41(11.26)	16(10.26)	
G3	444(85.38)	307(84.34)	137(87.82)	
G4	1(0.19)	1(0.27)		
GX	12(2.31)	10(2.75)	2(1.28)	
**Tumor residual**				
No macroscopic disease	92(17.69)	63(17.31)	29(18.59)	
1–10 mm	243(46.73)	165(45.33)	78(50)	
11–20 mm	34(6.54)	24(6.59)	10(6.41)	
>20 mm	104(20)	81(22.25)	23(14.74)	
Not available	47(9.04)	31(8.52)	16(10.26)	
**Venous invasion**				
No	54(10.38)	37(10.16)	17(10.9)	
Yes	78(15)	55(15.11)	23(14.74)	
Not available	388(74.62)	272(74.73)	116(74.36)	
**Cancer status**				
Tumor free	119(22.88)	85(23.35)	34(21.79)	
With tumor	345(66.35)	244(67.03)	101(64.74)	
Not available	56(10.77)	35(9.62)	21(13.46)	
**Site**				
Bilateral	372(71.54)	251(68.96)	121(77.56)	
Left	65(12.5)	50(13.74)	15(9.62)	
Right	56(10.77)	44(12.09)	12(7.69)	
Not available	27(5.19)	19(5.22)	8(5.13)	
**Race**				
American Indian or Alaska Native	3(0.58)	1(0.27)	2(1.28)	
Asian	16(3.08)	10(2.75)	6(3.85)	
Black or African American	22(4.23)	13(3.57)	9(5.77)	
Native Hawaiian or other Pacific Islander	1(0.19)	1(0.27)		
White	457(87.88)	325(89.29)	132(84.62)	
Not available	21(4.04)	14(3.85)	7(4.49)	
**Ethnicity**				
Hispanic or Latino	10(1.92)	5(1.37)	5(3.21)	
Not available	201(38.65)	143(39.29)	58(37.18)	
Not Hispanic or Latino	309(59.42)	216(59.34)	93(59.62)	

### 3.2. Determination of 21 methylation sites signature

**Figure 2.**
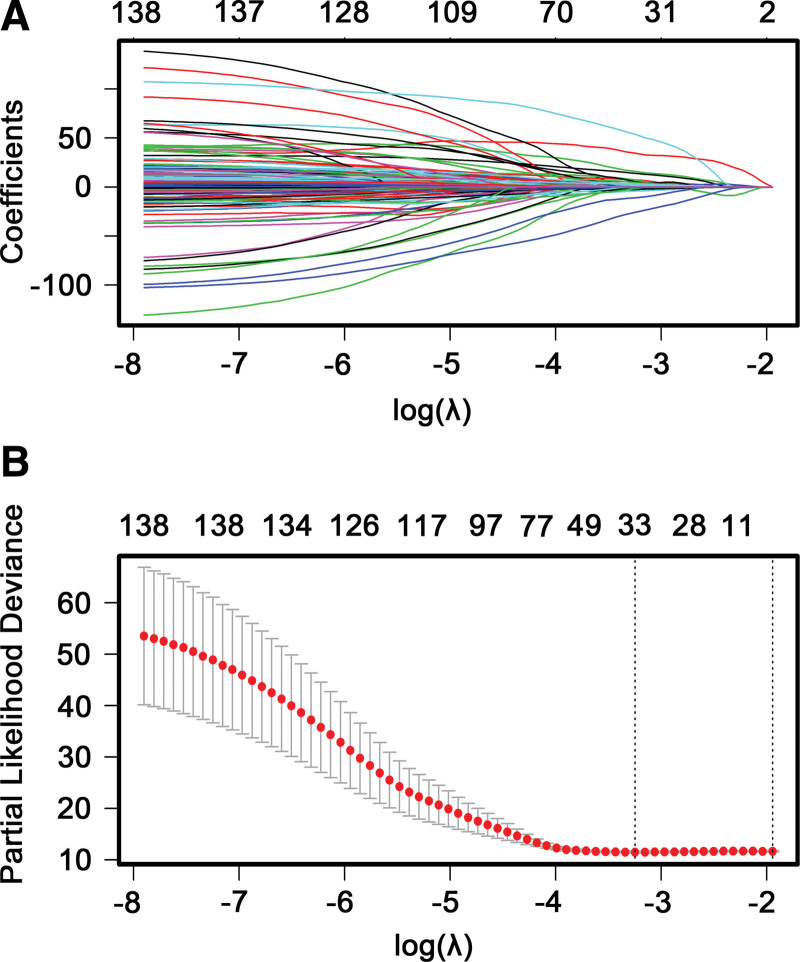
Candidate methylation sites selection on the basis of the LASSO Cox regression model. (A) 10-fold cross-validation for tuning parameter selection in the LASSO tool through minimum criteria (the 1-SE criteria). (B) LASSO coefficient profiles of the 138 methylation sites. A coefficient profile plot was created against log (lambda) sequence. Vertical line was implemented at the value selected by using 10-fold cross-validation, where optimal lambda resulted in 33 non-zero coefficients. LASSO = least absolute shrinkage and selection operator.

**Figure 3.**
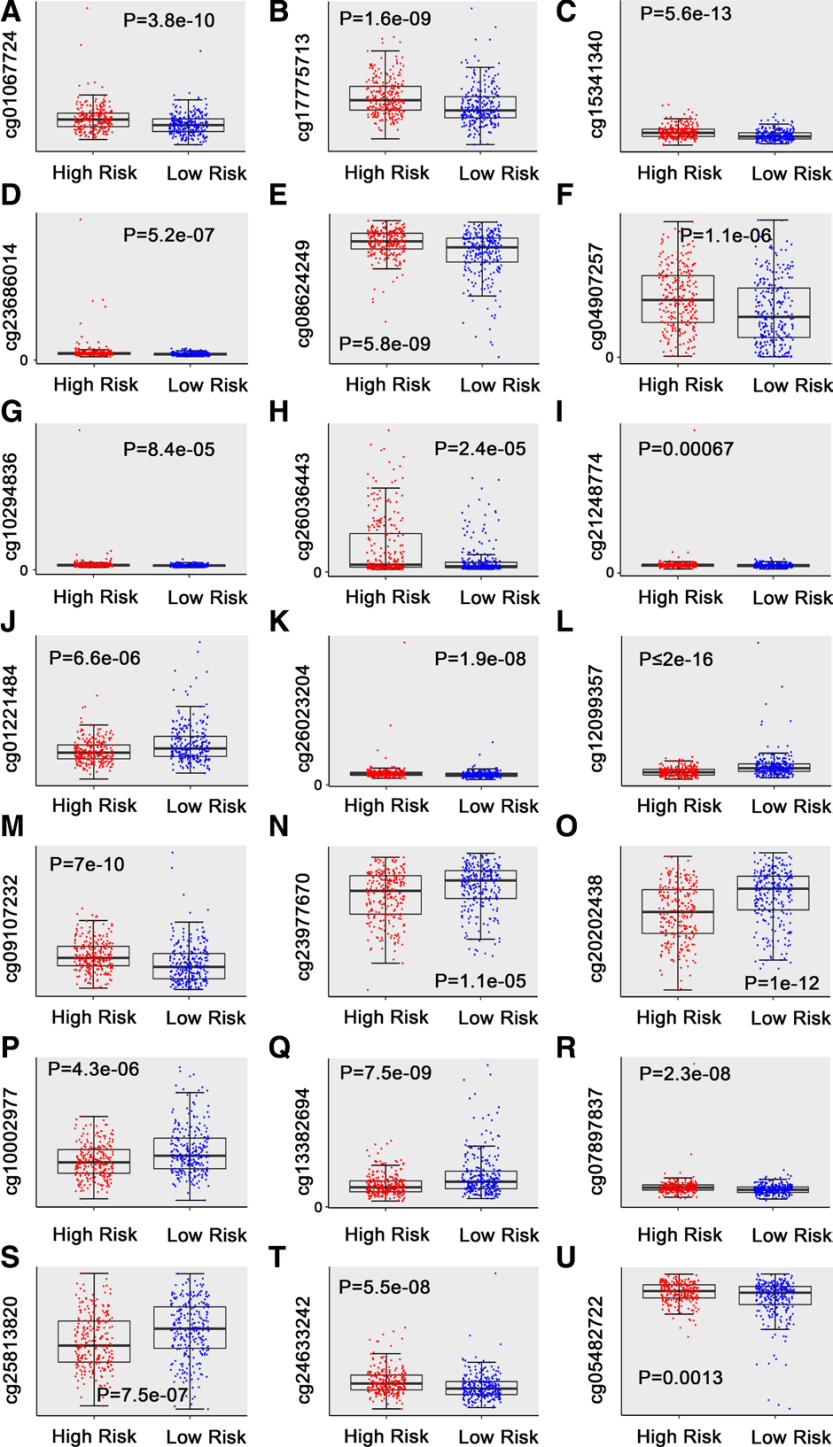
Boxplots of methylation β values against risk group in the entire TCGA dataset. “High Risk” and “Low Risk” refer to the high-risk and low-risk groups, respectively. The median risk score was applied as a cutoff. Y-axis stands for the β-value of 21-DNA methylation sites respectively. The differences between the 2 groups were weighed by Mann–Whitney *U* test. TCGA = the cancer genome atlas.

A total of 939 differentially methylated sites were screened from dead and alive cohorts, which were used for univariate Cox proportional hazard regression analysis, totally 138 DNA methylation sites was closely involved in stage III to IV OC patients’ OS (*P* < .01) (see Table S1, http://links.lww.com/MD/I377, Supplemental Digital Content, which show HR and 95% CIs as well as *P* values of 939 methylation sites based on univariate Cox regression analysis). Subsequently, the above 138 DNA methylation sites were mapped to LASSO Cox regression tool. After annotation, 33 methylation sites were screened as the candidate prognosis sites (*P* < .01) (Fig. 2A and B) for performing multivariate Cox proportional hazard regression. In the multivariate analysis, 21 methylation sites (*P* < .01) were finally selected for the construction of the DNA methylation predictor for OS of stage III to IV OC. Next, the 21 methylation sites were used to construct the optimal risk-score formula. Risk score = 24.98*cg06777274 + 1.03*cg17775713 + 24.58*cg15341340 + 7.37*cg23686014 + 3.58*cg08624249 + 0.68*cg04907257 + 2.38*cg10294836 + 1.34*cg26036443 + 11.07*cg21248774 - 48.07*cg01221484 + 19.66*cg26023204 - 11.94*cg12099357 - 10.26*cg09107232 - 1.13*cg23977670 - 0.73*cg20202438 - 26.08*cg10002977 - 3.21*cg13382694 + 76.21*cg07897837 - 0.90*cg25813820 + 14.72*cg24633242 + 10.19*cg05482722. The results showed that the hypermethylation levels of cg06777274, cg17775713, cg15341340, cg23686014, cg08624249, cg04907257, cg10294836, cg26036443, cg21248774, cg26023204, cg07897837, cg24633242, cg05482722 had a poor prognosis for OS, whereas the hypomethylation levels of cg01221484, cg12099357, cg09107232, cg23977670, cg20202438, cg10002977, cg13382694, cg25813820 had a dismal prognosis for OS (Fig. 3; see Fig. S1, http://links.lww.com/MD/I374, Supplemental Digital Content, which shows Boxplots of 21 methylation β values against risk group in OV-AU project). These results indicated the establishment of 21 methylation sites signature.

### 3.3. Interplays between 21 DNA methylation signature and stage III-IV OC patients’ OS in the internal validation, external validation and entire TCGA set

**Figure 4.**
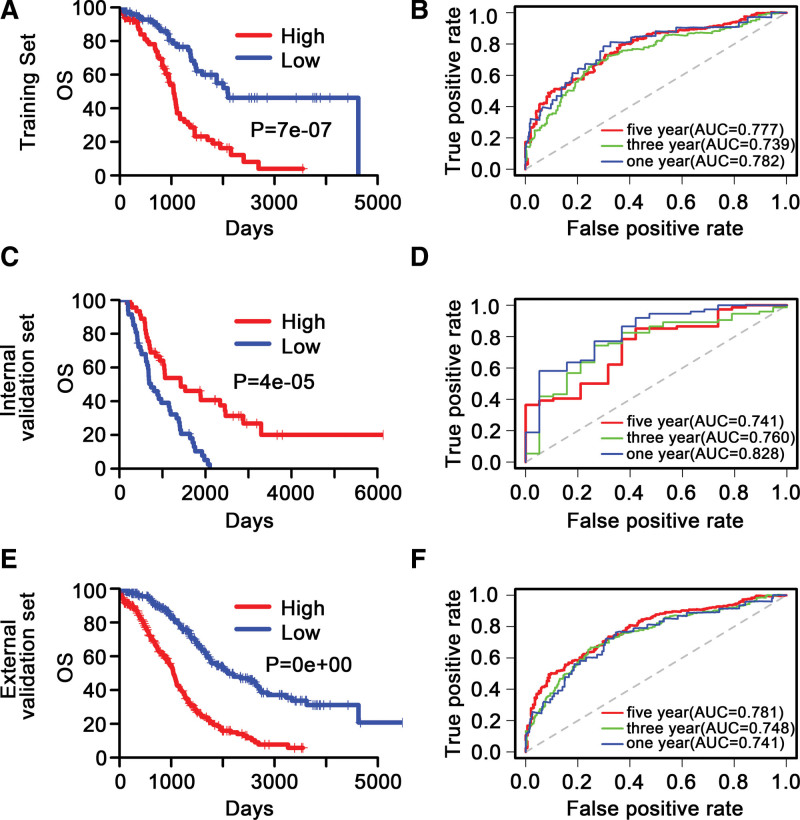
Kaplan–Meier and ROC analysis of patients with stage III to IV OC in the internal validation, external validation and entire TCGA set. (A, C, E) Kaplan–Meier analysis with 2-sided log-rank test was implemented to assess the differences in OS between the low-risk and high-risk cluster stage III to IV OC patients. (B, D, F) 1-, 3-, 5-year ROC curves of the 21-DNA methylation-based signature were adopted to assess the value of predicting the OS of stage III to IV OC patients. “High” and “Low” stood for the high risk score group and low risk score group, respectively. The median risk score was set as a cutoff. OC = ovarian cancer, OS = overall survival, ROC = relative operating characteristic curve, TCGA = the cancer genome atlas.

Samples were separated into the low- versus high- risk cohort in accordance to the 21-DNA methylation-related classifier. Kaplan–Meier survival analysis was employed to distinguish the difference in OS between the 2 cohorts. The patients in high- risk cohort had a significantly poor OS in internal validation set (*P* = 7e-07) (Fig. 4A), similar outcomes were shown in external validation set (*P* = 4e-05) (Fig. 4C) and entire TCGA set (*P* = 0e + 00) (Fig. 4E). These results suggested that our signature can effectively distinguish patients with good and poor prognosis.

### 3.4. Exploration of the predictive capacity of the 21-DNA methylation signature with ROC analysis

The power of the 21-DNA methylation-related classifier for stage III-IV OC patients’ OS was examined with a time-dependent ROC curve. The AUC of the 21-DNA methylation-related classifier at 1, 3, 5 years in internal validation set were 0.782, 0.739, 0.777, respectively (Fig. 4B). A good ability was also exhibited in external validation set (0.828, 0.760, 0.741) (Fig. 4D) and entire TCGA set (0.741, 0.748, 0.781) (Fig. 4F), exhibiting that 21-DNA methylation-related biomarker had a great power for predicting OS of OC patients.

**Figure 5.**
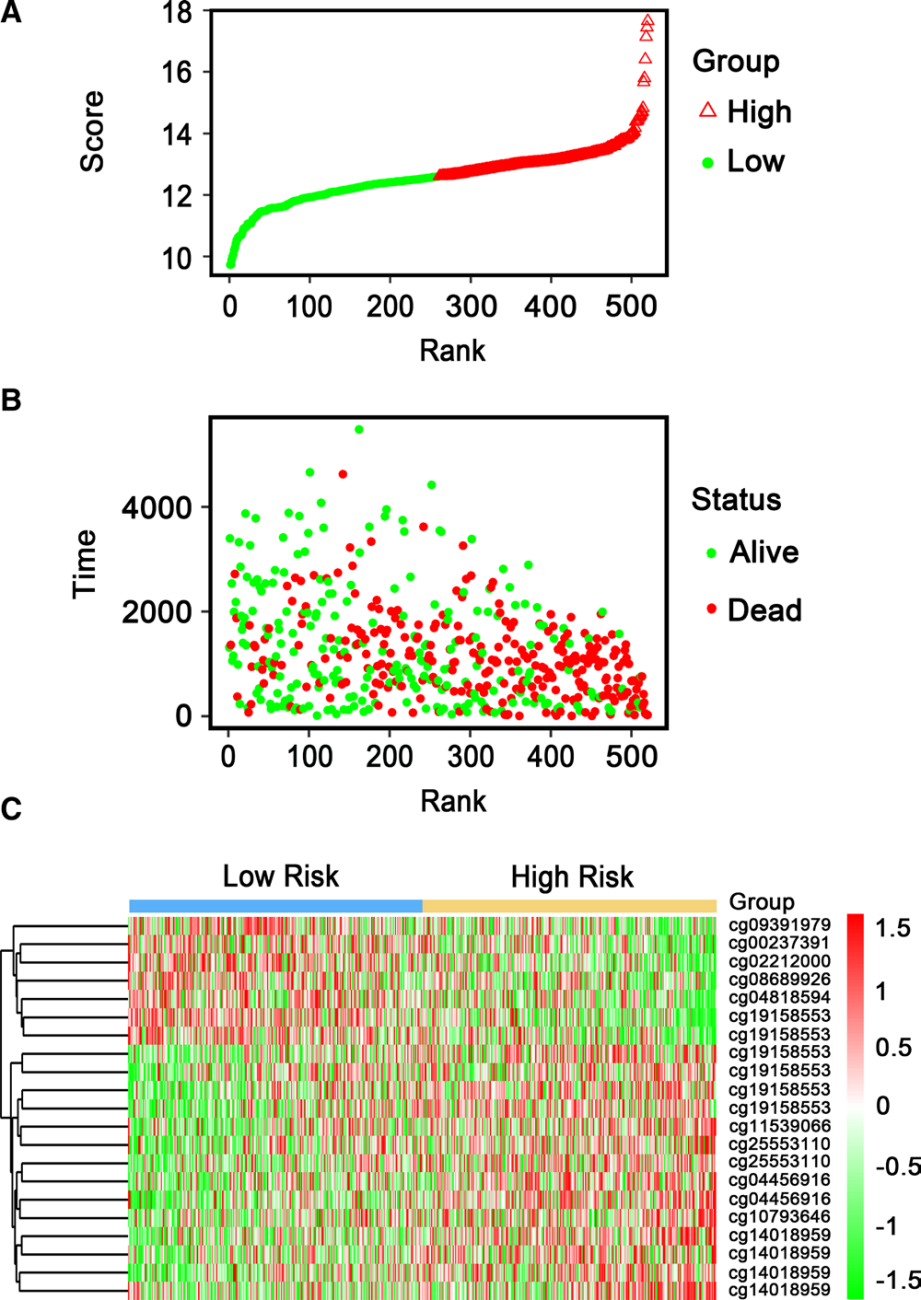
Methylation risk score analysis of 520 stage III to IV OC in the entire TCGA dataset. (A) Methylation risk score distribution against the rank of risk score. Median risk score functioned as the cutoff point. (B) Survival status of stage III to IV OC patients against the rank of risk score. (C) Heatmap of 21 methylation sites expression profiles of stage III to IV OC patients. OC = ovarian cancer, TCGA = the cancer genome atlas.

Next, samples were ranked on the strength of their risk scores (Fig. 5A), the dotplot was implemented in the light of recurrence status (Fig. 5B). We discovered that the high-risk cohort created a worse OS than that in the low-risk cluster. Heatmap of 21 methylation sites distribution on the basis of risk score was presented in Figure 5C, which generated a similar performance to our previous boxplot (see Fig. S2, http://links.lww.com/MD/I375, Supplemental Digital Content, which shows methylation risk score analysis of 93 stage III–IV OC patients in OV-AU project; see Table S2, http://links.lww.com/MD/I378, Supplemental Digital Content, which show 21 DNA methylation signature-associated biological pathways).

Finally, subgroup analysis was performed on the strength of several clinical covariates which consisted of age, stage, tumor residual and cancer status. A great ability of the 21-DNA methylation-related classifier was shown in most sub-group (see Figs. S3–S6, http://links.lww.com/MD/I376, Supplemental Digital Content, which shows Kaplan–Meier and ROC analysis of patients with stage III–IV OC in sub-groups according to age, stage, tumor residual and cancer status, respectively.). These findings demonstrated that our DNA methylation signature yield a high predictive power.

### 3.5. Identification of the 21 DNA methylation classifier-related biological pathways

**Figure 6.**
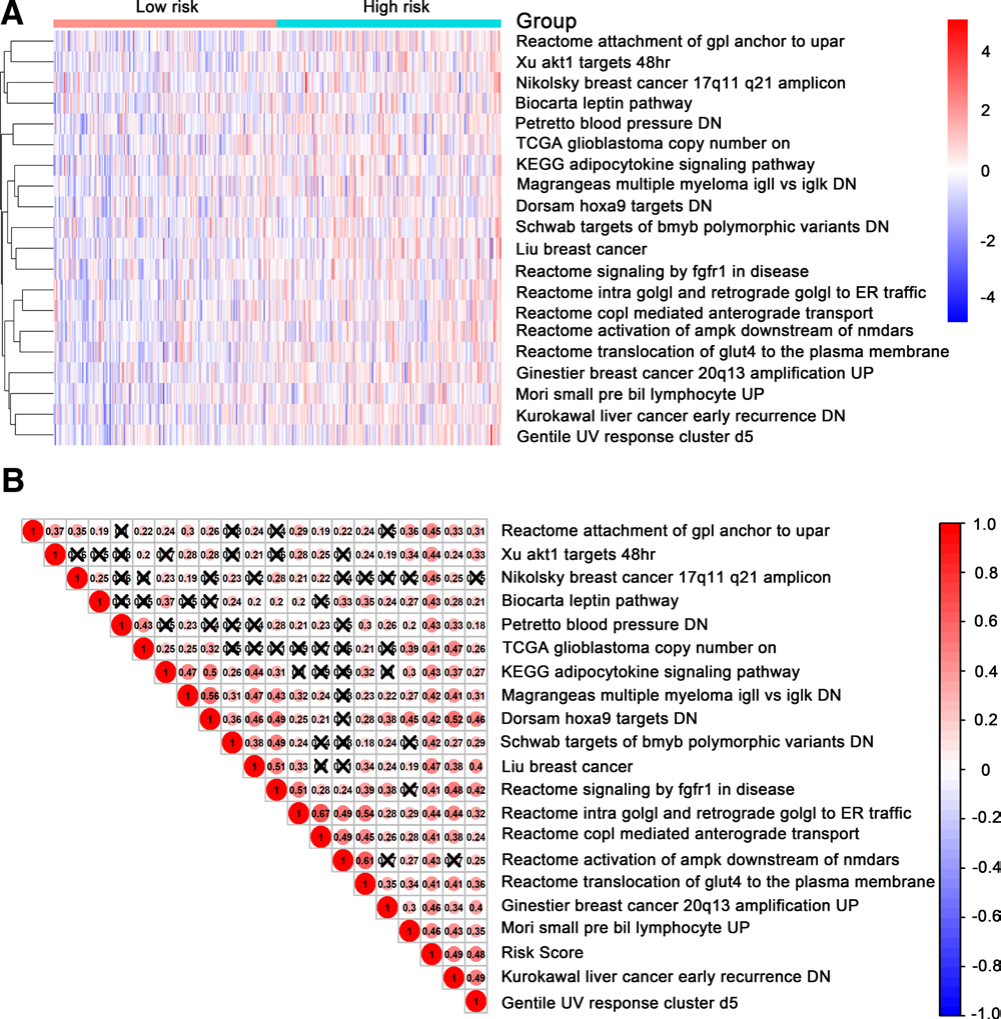
Identification of the 21 methylation signature-relevant biological pathways. (A) Heatmap of top 20 enriched pathways associated with high risk group. (B) Association graph between risk scores and top 20 pathways.

The median score was employed as the cutoff to stratify samples into low- and high-risk group. Top 20 pathways which were more triggered in the high-risk cases than that in low-risk cases were presented in Figure 6A. The same trend was further proved between enriched pathways and DNA methylation risk score (Fig. 6B).

### 3.6. Nomogram construction

**Figure 7.**
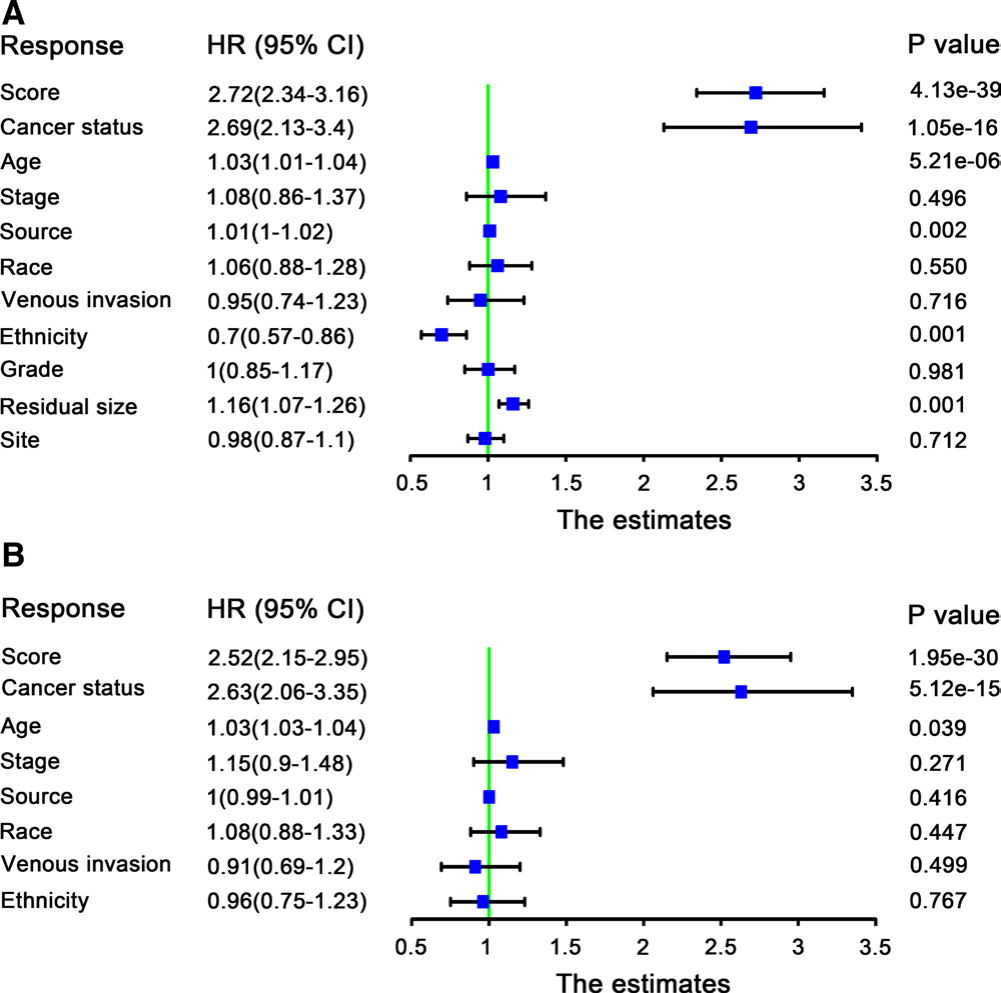
Forest plot summary of analysis of OS. (A) Univariable Cox analysis of the risk score and other clinical factors. (B) Multivariable Cox analysis of the risk score and other clinical factors. The blue squares on the transverse lines represent the hazard ratio (HR), and the black transverse lines represent 95% CI. OS = overall survival.

**Figure 8.**
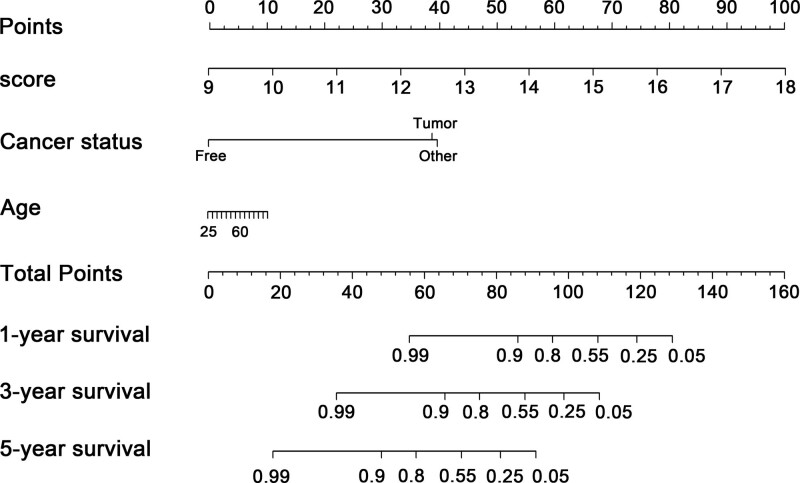
21-DNA methylation-associated nomogram for the prediction of stage III-IV OC patients’ OS. The nomogram was constructed in the entire TCGA cohort based on the DNA methylation risk score, age as well as cancer status. OC = ovarian cancer, OS = overall survival, TCGA = the cancer genome atlas.

**Figure 9.**
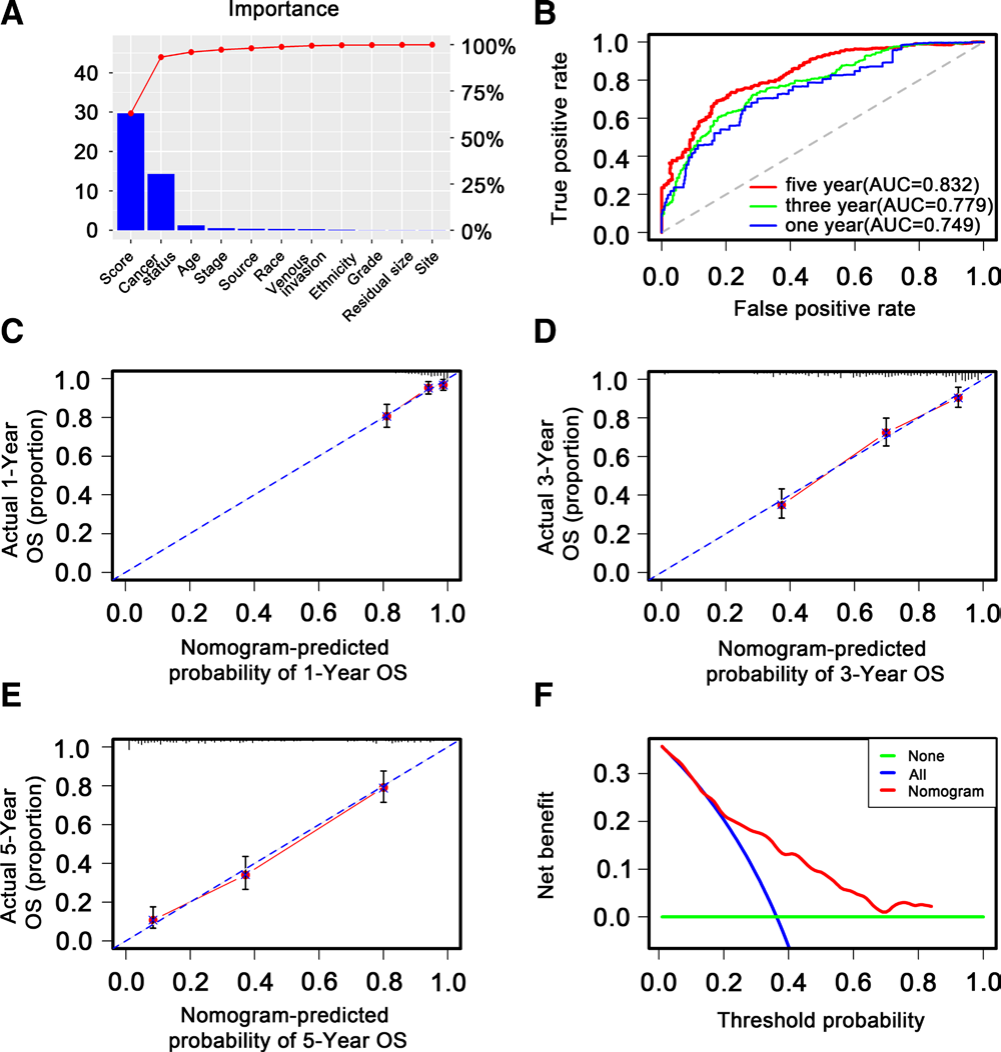
Validation of the DNA methylation gene-related nomogram in the entire TCGA dataset. (A) The higher the bar chart, the greater the percentage. (B) 1-, 3-, 5-year receiver operating characteristic curves for the metabolic gene-related nomogram. (C, D, E) referred to the 1-, 3-, 5-year nomogram calibration curves, respectively. The closer the dotted line fitted to the ideal line, the better the predictive value of our nomogram. (F) The DCA for the nomogram. The net benefit was plotted versus the threshold probability. The red line represented the nomogram. The blue line represented the treat-all and the green line represented the treat-none. DCA = decision curve analysis, TCGA = the cancer genome atlas.

To explore whether the 21-DNA methylation-related signature was an independent classifier for stage III-IV OC patients’ OS, univariate and multivariate Cox tool was executed on the strength of methylation-related risk score and a few clinical covariates. HRs showed that the 21-DNA methylation-related classifier was tightly related to stage III-IV OC patients’ OS (*P* < .001, HR 2.15, 95% CI 2.15–2.50) (Table 2) (Fig. 7), manifesting that the 21-DNA methylation-related signature functioned as an independent prognosis classifier. To predict stage III to IV OC patients’ OS on the basis of a quantitative strategy, we developed a nomogram (Fig. 8) according to risk score cancer status, as well as age. The significance between the 21-DNA methylation-based factor and the conventional clinical covariates was exhibited in (Fig. 9A). The power of the nomogram was tested on the strength of C-index (0.818, 95%CI: 0.778–0.864), AUC (1, 3, 5-year: 0.749, 0.779, 0.832, respectively) (Fig. 9B) and calibration plot (Fig. 9C–E), manifesting a strong power of the model. Besides, decision curve analysis proved that the nomogram generated more important clinical utilization for prognostic prediction of stage III to IV OC than that in treat all or treat none cluster. Net benefit was achieved for stage III to IV OC patients in 3-year recurrent risks (Fig. 9F). The result proved that our methylomics-based nomogram generated a great capacity and may have potential for clinical utilization.

**Table 2:** Univariate and multivariate Cox regression analysis results on the basis of DNA methylation risk score and other clinical factors.

	Univariate Cox analysis				Multivariate Cox analysis			
Characteristics	HR	HR.95L	HR.95H	*P* value	HR	HR.95L	HR.95H	*P* value
Score	2.718282	2.340085	3.157602	4.13E-39	2.517934	2.15026	2.948477	1.95E-30
Cancer status	2.690104	2.129446	3.398377	1.05E-16	2.629841	2.064107	3.350632	5.12E-15
Age	1.025523	1.014464	1.036701	5.21E-06	1.031618	1.019877	1.043497	.039473
Stage	1.084461	0.858714	1.369556	.495945	1.152149	0.895167	1.482904	.271374
Tissue source site	1.011779	1.004328	1.019286	.001901	1.003807	0.994653	1.013046	.416273
Race	1.059866	0.875764	1.28267	.550335	1.082994	0.881884	1.329967	.446834
Venous invasion	0.953968	0.740202	1.229469	.715819	0.910171	0.69295	1.195485	.498693
Ethnicity	0.701931	0.570451	0.863715	.000824	0.963741	0.754723	1.230645	.767156
Grade	0.99803	0.851449	1.169845	.980584	0.989464	0.841468	1.16349	.898046
Tumor residual disease	1.161109	1.06712	1.263377	.000524	1.00551	0.916168	1.103563	.907863
Site	0.978786	0.873539	1.096714	.711814	0.994693	0.88535	1.117539	.928634

HR = hazard ratio.

## 4. Discussion

In this study, TCGA databases and ICGC database were used to explore the DNA methylation hallmark for OS in stage III to IV OC. finally, we identified a signature which contained 21 DNA methylation sites by combining differential methylation analysis, Cox regression analysis, ROC analysis, and Kaplan–Meier analysis. The above 21 DNA methylation sites were corresponding to 24 genes (ME2, TF, DNASE2, TFPI2, C20orf117, ADCY2, DYRK1B, EHD2, SFPQ, MCM7, AP4M1, C1orf131, GNPAT, AKTIP, UTP14C, ALG11, ZNF584, FAM76A, MYNN, H19, NPPA, SOD2, ZNF652, MPP1). Interestingly, previous studies have shown that most of these 24 genes were associated with cancer, respectively. For instance, Rita et al found that combination of 2-methoxyestradiol (2-ME2) and eugenol were involved in androgen independent prostate cancer cells.^[20]^ Xu et al identified cancer subtypes from miRNA-TF-mRNA regulatory networks and expression Data.^[21]^ Marek et al indicated that Human OC cells may be eradicated by transgenic expression of recombinant DNASE1, DNASE1L3, DNASE2, and DFFB controlled by EGFR promoter.^[22]^ Dong et al suggested that hypermethylation of TFPI2 was correlated with cervical cancer incidence in the Uygur and Han populations of Xinjiang, China.^[23]^ Yu et al revealed that SPARCL1, SHP2, MSH2, e-cadherin, p53, ADCY-2 and MAPK were prognosis-associated in colorectal cancer.^[24]^ Chen et al reported that DYRK1B overexpression was associated with breast cancer growth and a poor prognosis.^[25]^ Yang et al demonstrated that EHD2 played an important role in migration and invasion of human breast cancer cells.^[26]^ NEAT1_2-SFPQ axis mediated cisplatin resistance in liver cancer cells in vitro.^[27]^ MCM7 promoted cancer progression via cyclin D1-dependent signaling and served as a prognostic hallmark for patients with hepatocellular cancer.^[28]^ Stabilization of FASN by ACAT1-mediated GNPAT acetylation promoted lipid metabolism and hepatocarcinogenesis.^[29]^ It has recently been suggested that UTP14c are expressed in 80% of OCs.^[30]^ Khakpour et al suggested that ZNF584 was involved in breast cancer.^[31]^ MYNN and TERC gene polymorphisms were associated with bladder cancer in a Turkish population.^[32]^ LncRNA H19 promoted lung cancer proliferation and metastasis via inhibiting miR-200a function.^[33]^ Strong SOD2 expression and HPV-16/18 positivity were independent events in cervical cancer.^[34]^ Co-expression of the androgen receptor and the transcription factor ZNF652 was related to prostate cancer outcome.^[35]^ The result exhibited that the 18 of the 24 genes related to the 21 sites played key roles in development of cancer. We speculated that the above 18 of the 24 genes may be involved in the prognosis of stage III to IV OC patients.

Accumulating studies performed nomograms to elevate prognostic robustness for clinical result via incorporating a few independent clinical factors into a single quantitative risk probability. For instance, Amita et al revealed preoperative nomograms combining magnetic resonance imaging and spectroscopy for predicting insignificant prostate carcinoma.^[36]^ Lee et al showed a prognosis nomogram to predict OS in patients with platinum-sensitive recurrent OC.^[37]^ Our study was the first to execute the transformative utility combining clinical factors and methylation predictor for application beyond simple classification into field of individualized stage III to IV OC patients’ OS. The outcome implied great capacity of our nomogram for predicting stage III to IV OC patients’ OS in the clinical routine, which made our outcome more reliable.

The LASSO is on the basis of shrinkage estimation and has been frequently applied in the statistical field. For example, a previous study reported the application of ayesian LASSO for genomic selection in French holstein and montbéliarde breeds.^[38]^ A research revealed parsimonious and robust multivariate calibration via rational function of LASSO and Rational Function Elastic Net.^[39]^ The LASSO whose mean squared error is smaller than that in conventional approaches can overcome the multicollinearity issue and implement the function of overall variable selection and coefficients shrink.^[40–42]^ In our study, LASSO Cox regression model was employed to explore the candidate DNA methylation sites associated with stage III to IV OC patients’ OS that excluded the factors between univariate and multivariate Cox analysis for excluding the interference of the possible multicollinearity in this study. That is to say, LASSO COX regression tool improved the predictive accuracy of the 21-DNA methylation-related classifier.

Several limitations existed in our study. Firstly, the number of the stage III-IV OC patients of our external validation set was limited and prospective study with a more samples was needed to validate the value of the 21-DNA methylation signature. In addition, more clinical factors should be mapped in the external validation set to improve the reliability of the DNA methylation-related predictive model. Besides, the nomogram was developed through retrospective data obtained from TCGA database, which may have hazard of selection bias.

In conclusion, in spite of the limitations mentioned above, there are still valuable implications in this comprehensive high throughput data analysis, which identified 21-DNA methylation signature for predicting stage III to IV OC patients’ OS via combining differential methylation analysis, Cox regression analysis, ROC analysis, and Kaplan–Meier analysis. Besides, we constructed a nomogram that integrated the 21-DNA methylation-related signature and several clinical covariates to strengthen the predicted accuracy in a quantitative method for prognosis of stage III to IV OC. The result indicated that our nomogram yielded a strong robustness for predicting OS of stage III to IV OC.

## Author contributions

**Conceptualization:** Xuan Wei, Kexi Mao.

**Data curation:** Xuan Wei.

**Formal analysis:** Xuan Wei.

**Funding acquisition:** Xuan Wei, Kexi Mao.

**Investigation:** Wencheng Hu, Kexi Mao.

**Methodology:** Xuan Wei, Wencheng Hu, Kexi Mao.

**Project administration:** Wencheng Hu, Kexi Mao.

**Resources:** Wencheng Hu, Kexi Mao.

**Software:** Xuan Wei.

**Supervision:** Wencheng Hu, Kexi Mao.

**Validation:** Wencheng Hu, Kexi Mao.

**Visualization:** Wencheng Hu, Kexi Mao.

**Writing – original draft:** Xuan Wei.

**Writing – review & editing:** Wencheng Hu, Kexi Mao.

## Supplementary Material












