# Supplementary material for: A methylomics-associated nomogram predicts the overall survival risk of stage III to IV ovarian cancer
Source: Medicine (Baltimore). 2023 Feb 3;102(5):e32766. doi: 10.1097/MD.0000000000032766 (PMC9901957; doi:10.1097/MD.0000000000032766)

Figure S1. Boxplots of 21 methylation β values against risk group in OV-AU project. "High Risk" and "Low Risk" represent the high-risk and low-risk group, respectively. The median risk score was taken as a cutoff. Y-axis represent the β-value of 21-DNA methylation sites respectively.

Figure S1

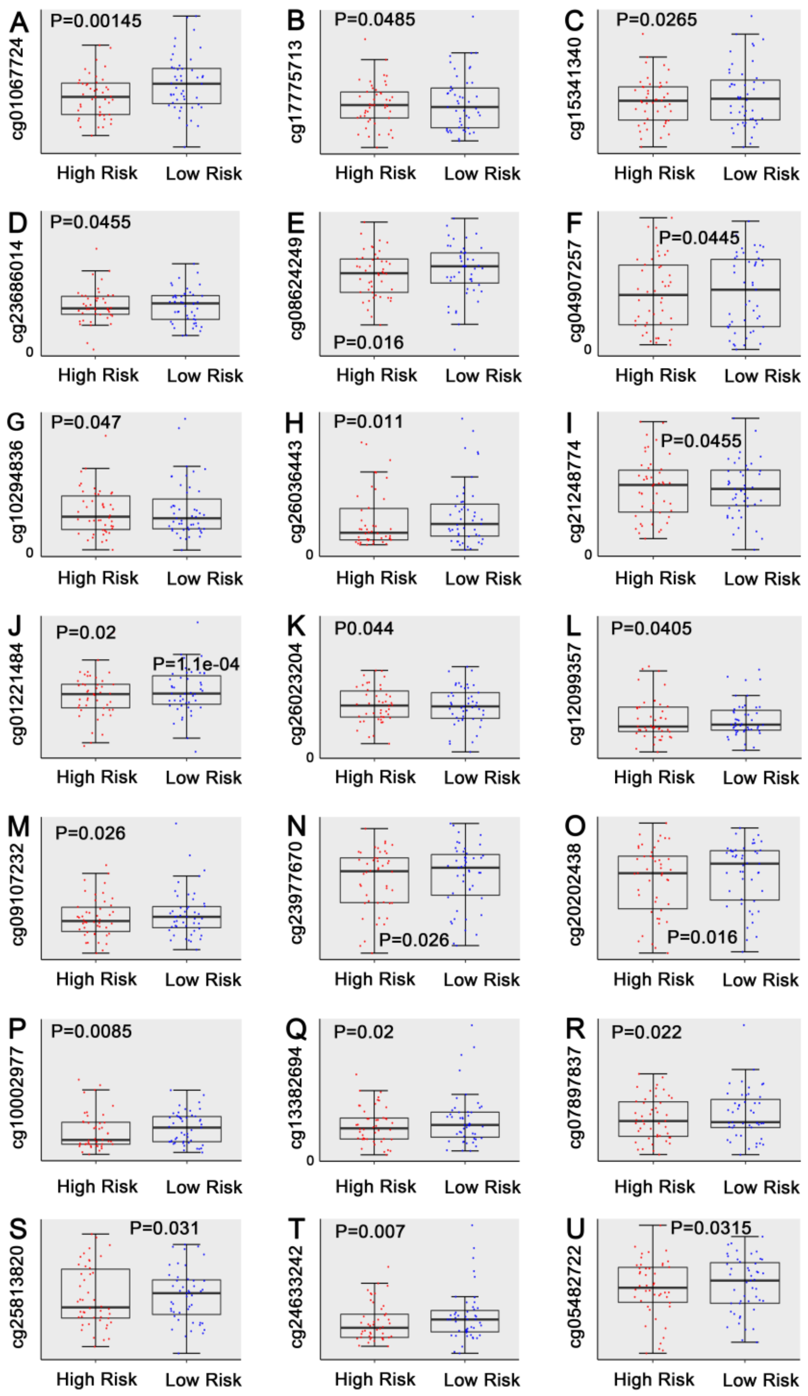

Supplement: Supplementary file 2 [file medi-102-e32766-s002.pdf]
