# Supplementary material for: A methylomics-associated nomogram predicts the overall survival risk of stage III to IV ovarian cancer
Source: Medicine (Baltimore). 2023 Feb 3;102(5):e32766. doi: 10.1097/MD.0000000000032766 (PMC9901957; doi:10.1097/MD.0000000000032766)

Figure S2. **Methylation risk score analysis of 93 stage III-IV OC patients in OV-AU project. (A)** Methylation risk score distribution against the rank of risk score. Median risk score was the cut-off point. **(B)** Recurrence status of stage III-IV OC patients. **(C)** Heatmap of 21 methylation sites expression profiles of stage III-IV OC patients.

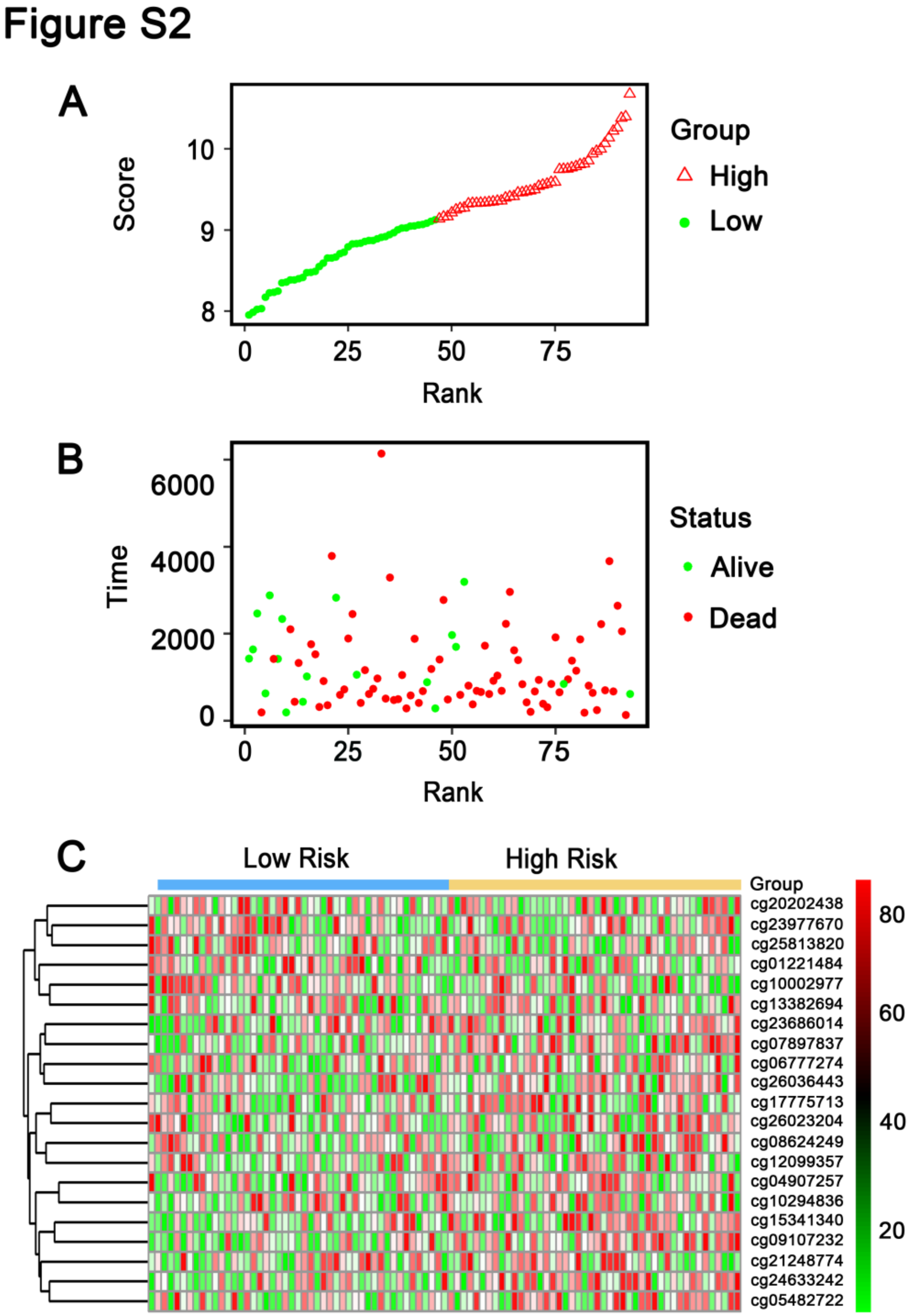

Supplement: Supplementary file 3 [file medi-102-e32766-s003.pdf]
