# Supplementary material for: A methylomics-associated nomogram predicts the overall survival risk of stage III to IV ovarian cancer
Source: Medicine (Baltimore). 2023 Feb 3;102(5):e32766. doi: 10.1097/MD.0000000000032766 (PMC9901957; doi:10.1097/MD.0000000000032766)

Figure S3. Kaplan-Meier and ROC analysis of patients with stage III-IV OC in sub-groups according to age, respectively. (A, B) Age less 60 years sub-group. **(C, D)** Age over 60 years sub-group. “High” and “Low” stood for the high risk score group and low risk score group, respectively. The median risk score was set as a cutoff.

Figure S3

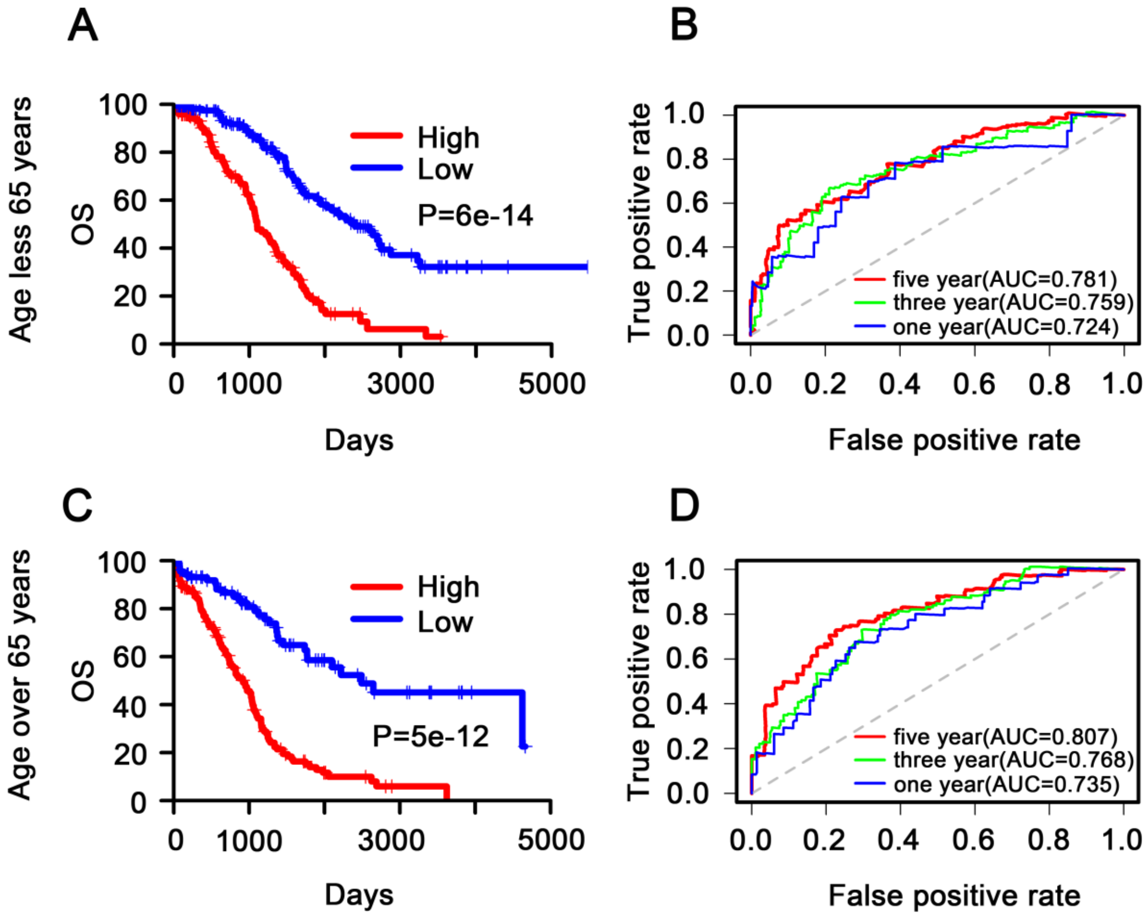

Figure S4. Kaplan-Meier and ROC analysis of patients with stage III-IV OC in sub-groups according to stage, respectively. (A, B) Stage III sub-group. **(C, D)** Stage **IV** sub-group. “High” and “Low” represented the high risk score group and low risk score group, respectively. The median risk score was set as a cutoff.

Figure S4

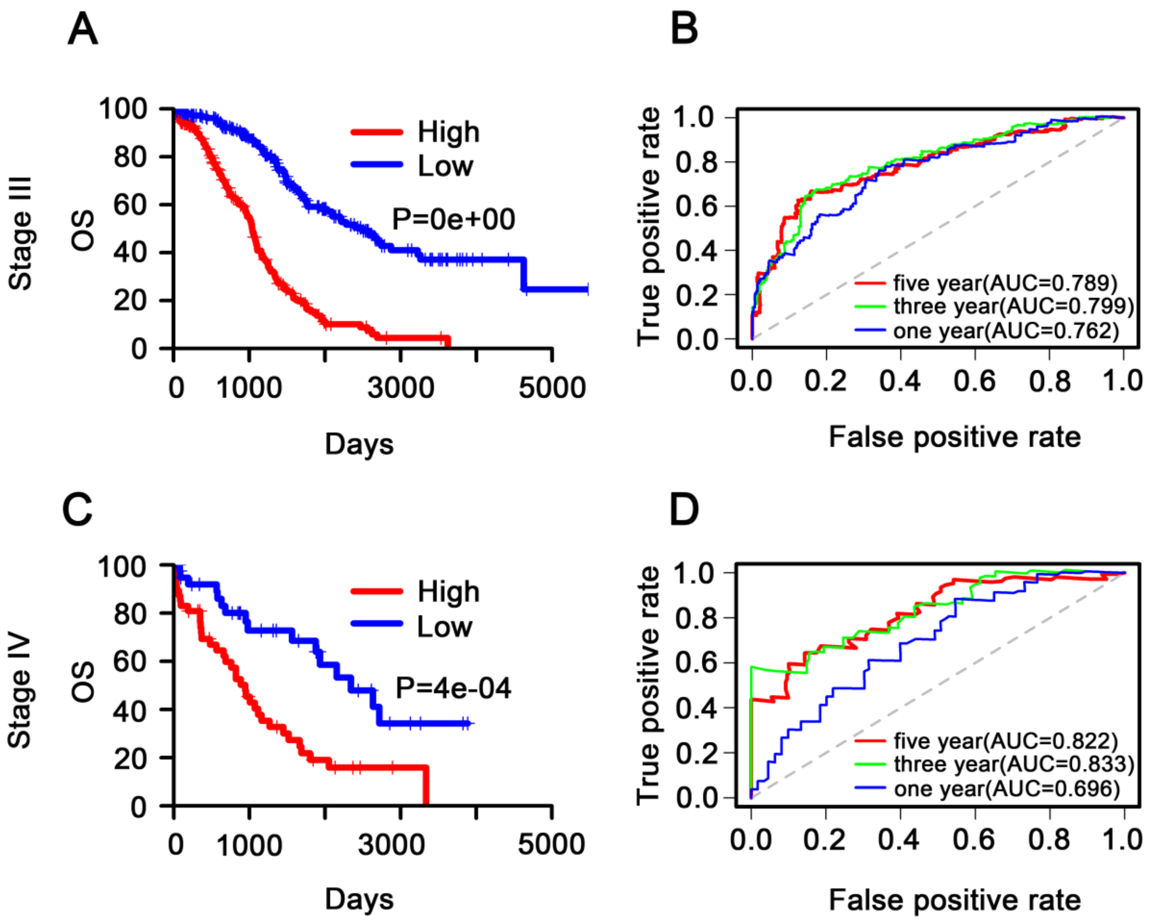

Figure S5. Kaplan-Meier and ROC analysis of patients with stage III-IV OC in sub-groups according to tumor residual, respectively. (A, B) Less 10mm group. **(C, D)** Over 10mm group. “High” and “Low” represented the high risk score group and low risk score group, respectively. The median risk score was set as a cutoff.

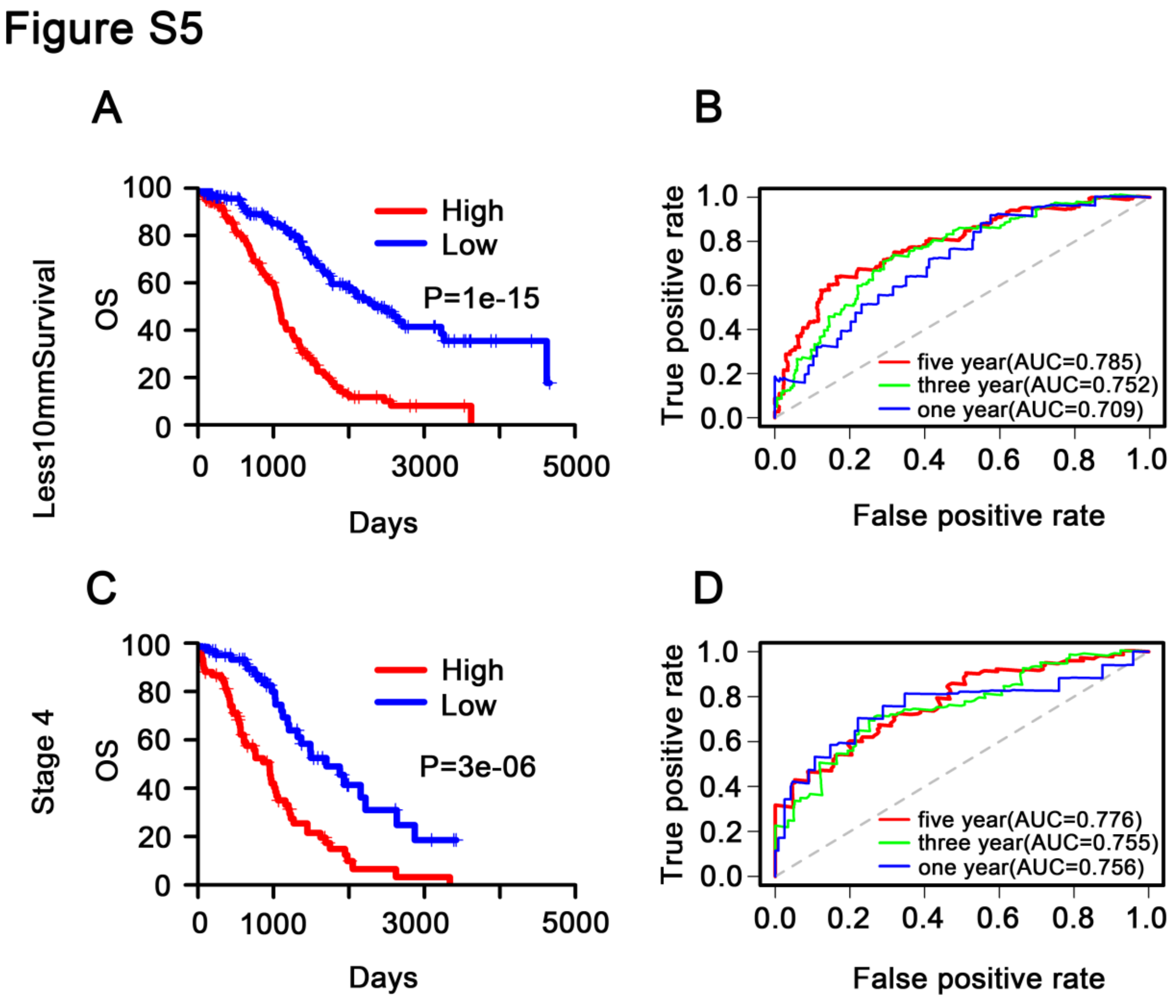

Figure S6. Kaplan-Meier and ROC analysis of patients with stage III-IV OC in sub-groups according to cancer status, respectively. (A, B) With tumor group. **(C, D)** Tumor-free group. "High" and "Low" represented the high risk score group and low risk score group, respectively. The median risk score was set as a cutoff.

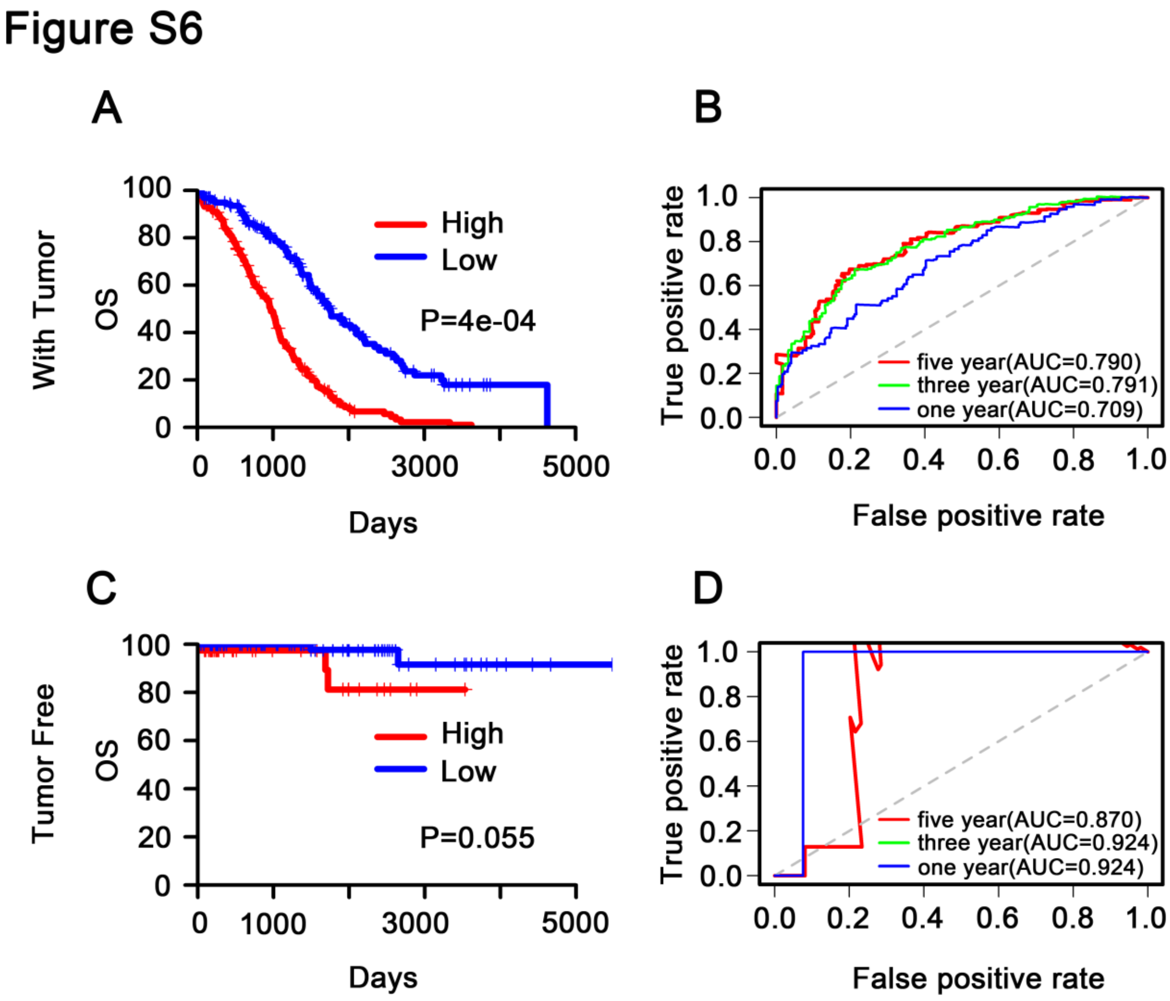

Supplement: Supplementary file 4 [file medi-102-e32766-s004.pdf]
